# Comparison of Amiodarone and Propafenone in Blanking Period after Radiofrequency Catheter Ablation in Patients with Atrial Fibrillation: A Propensity Score-Matched Study

**DOI:** 10.1155/2020/1835181

**Published:** 2020-06-26

**Authors:** RongDa Huang, JingJing Lin, KeZeng Gong, LiangLong Chen, Lin Fan, FeiLong Zhang, Yan Zhang, XueHai Chen, Zhe Xu

**Affiliations:** ^1^Department of Cardiology, Fuzhou First Hospital of Fujian Medical University, Fuzhou, Fujian, China; ^2^Department of Cardiology, Fujian Heart Medical Center, Fujian Medical University Union Hospital, Fujian Institute of Coronary Heart Disease, Fuzhou, Fujian, China

## Abstract

**Background:**

Amiodarone and propafenone are commonly used to maintain sinus rhythm in patients with atrial fibrillation (AF). However, it is not known which one is better in reducing early recurrence (ER) during the blanking period (the first three months after catheter ablation).

**Objective:**

To compare the efficacy and safety of amiodarone and propafenone in reducing ER during the blanking period after radiofrequency catheter ablation (RFCA) in AF patients.

**Materials and Methods:**

A total of 694 patients who underwent their first RFCA between May 2014 and May 2018 were enrolled in this retrospective study. Subsequently, 202 patients were excluded according to the exclusion criteria. The remaining 492 patients were divided into two groups based on the choice of antiarrhythmic drugs (AADs) (amiodarone or propafenone) in the blanking period. The primary outcomes were incidence of ER and AAD-associated adverse effects during the blanking period after RFCA. Propensity score matching (PSM) analyses were used to compare the outcomes of the two groups while controlling for confounders.

**Results:**

Among the 492 patients who took AADs in the blanking period (187 amiodarone and 305 propafenone), PSM selected 135 unique pairs of patients with similar characteristics. Amiodarone was associated with a lower ER incidence rate (23.7% versus 48.9%, *p* < 0.001) and a similar rate of AAD-associated adverse effects (2.1% versus 1.5%, *p* = 0.652). Treatment with amiodarone in the blanking period was significantly associated with a lower ER incidence rate compared to treatment with propafenone (HR = 0.416, 95% CI 0.272–0.637, *p* < 0.001).

**Conclusions:**

Compared with propafenone, amiodarone was associated with a lower ER incidence rate, and they had similar rates of AAD-associated adverse effects. Treatment with amiodarone in the blanking period was shown to be more effective in reducing ER than propafenone.

## 1. Introduction

Radiofrequency catheter ablation (RFCA) using the technique of pulmonary vein isolation (PVI) is the first-line strategy for the treatment of symptomatic and drug-refractory atrial fibrillation (AF) [1, 2]. However, recurrences of AF, atrial flutter (AFL), and atrial tachycardia (AT) after initially successful catheter ablation are common. Early recurrence (ER) is defined as any atrial tachyarrhythmia (AF, AFL, and AT) lasting at least 30 s occurring during the first three months post ablation [3]. ERs within the first three months post PVI have been reported in up to 50% of patients [4]. It is currently believed that ER, unlike late recurrence (LR) (recurrence between three and 12 months after RFCA), is a transient phenomenon attributed to ablation-related inflammatory reactions and cardiac autonomic dysfunction [5, 6]. Since most ERs will return to sinus rhythm without intervention, the period of three months after RFCA is defined as the “blanking period.” Although ER does not always lead to LR during a long-term follow-up period, many studies have confirmed that ER is an independent risk factor associated with LR [7], which is a concern. Short-term application of antiarrhythmic drugs (AADs) in the blanking period can reduce the risk of ER effectively [7, 8]. However, it is not known which antiarrhythmic drug is more effective in reducing ER.

In patients with AF, both amiodarone and propafenone are recommended in guidelines to maintain sinus rhythm [7, 9, 10]. Propafenone was the preferred choice in practice due to its definite curative effect and fewer side effects. Amiodarone was not the preferred choice because of its extracardiac side effects, especially in long-term therapy [7, 9, 11]. Nick et al. found that amiodarone was the most effective drug in maintaining sinus rhythm; however, the rate of extracardiac adverse reactions was high in long-term application, and therefore, it was used as a second-line treatment in clinics [12]. However, it has been reported that amiodarone has particular anti-inflammatory and noncompetitive antisympathetic effects [13], which may help to reduce ER in the blanking period. Furthermore, the short-term use of amiodarone could be safer than the long-term application. We aimed to use propensity score matching (PSM) analyses to compare the efficacy and safety of amiodarone and propafenone in reducing ER during the blanking period after RFCA.

## 2. Materials and Methods

### 2.1. Study Population

**Figure 1:**
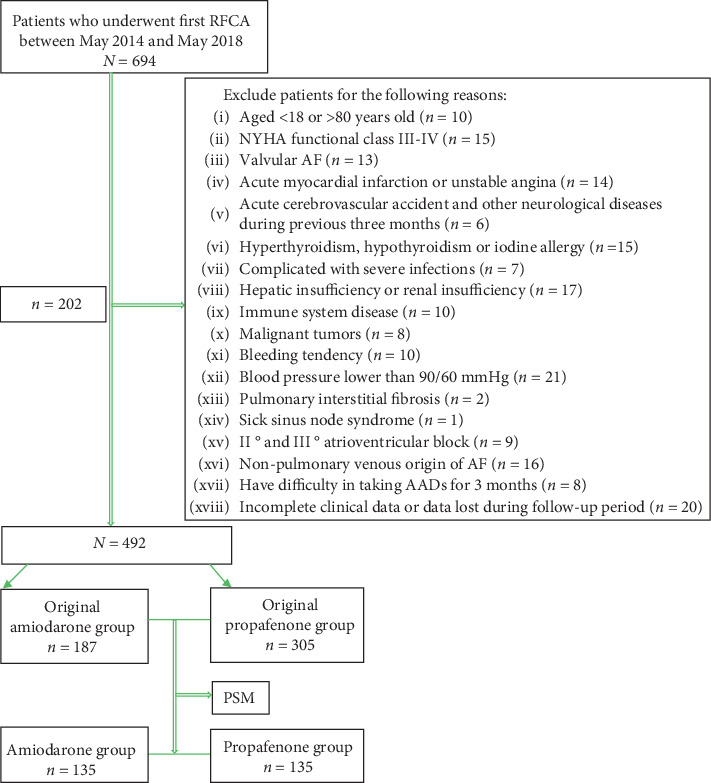
The flowchart for inclusion and exclusion.

A total of 694 patients with a diagnosis of AF who underwent their first RFCA between May 2014 and May 2018 at the Department of Cardiology of Fujian Union Medical College Hospital were enrolled in this retrospective observational study. Subsequently, 202 patients were excluded from analyses. They included (I) patients aged <18 or >80 years; (II) New York Heart Association (NYHA) functional class III−IV; (III) patients with valvular AF; (IV) patients with acute myocardial infarction or unstable angina; (V) patients with acute cerebrovascular accident and other neurological diseases during previous three months; (VI) patients with hyperthyroidism, hypothyroidism, or iodine allergy; (VII) patients complicated with severe infections; (VIII) patients with hepatic or renal insufficiency; (IX) patients with immune system disease or malignant tumors; (X) patients who have a tendency to bleed; (XI) patients whose blood pressure was lower than 90/60 mmHg; (XII) patients with pulmonary interstitial fibrosis; (XIII) patients with sick sinus node syndrome or II and III atrioventricular block; (XIV) patients with nonpulmonary venous origin of AF; (XV) patients who have difficulty in taking AADs for three months; (XVI) patients with incomplete clinical data or data lost during the follow-up period.  Figure 1 shows the flowchart for inclusion and exclusion.

### 2.2. Radiofrequency Catheter Ablation Procedure

The indication for RFCA was in accordance with the 2014 AHA/ACC/HRS Guideline for the Management of Patients with Atrial Fibrillation [14]. Of the 492 patients included in the study, 46 had taken *β*-blockers such as metoprolol or bisoprolol before ablation, eight had taken amiodarone, and 13 had taken propafenone. The longest time patients had taken amiodarone was three weeks, the shortest time was two days, and the median time was six days. Prior to RFCA, all antiarrhythmic drugs were stopped for at least five half-lives. Low-molecular-weight heparin or warfarin was administered from three weeks before ablation to the day of the ablation procedure.

The three-dimensional configuration of the left atrium was reconstructed by the CARTO 3 system, and the location of the bilateral pulmonary vein vestibule was marked. Bilateral pulmonary vein vestibular linear ablation was performed to isolate the pulmonary vein. The endpoint of RFCA was the disconnection of the pulmonary vein with the atrium, and the atrial blowout stimulation failed to induce <30 seconds of rapid atrial arrhythmia.

### 2.3. Treatment after RFCA

Amiodarone or propafenone was started immediately after the ablation procedure unless patients had contraindications or intolerance. The dosage of AADs was to be determined according to the guidelines [7, 14]. The initial dose of amiodarone was 600 mg/day in the first week after ablation, subsequently, 400 mg/day in the second week, and from the third week, kept maintenance dose of 200 mg/day, until three months. The dose of propafenone was 450 mg/day. Antiarrhythmic therapy continued during the follow-up period or until ER occurred. Oral anticoagulation routinely lasted for at least three months. Subsequent administration would depend on the risk stratification of stroke in accordance with the CHA2DS2–VASc. Medications including beta-blockers, angiotensin-converting enzyme inhibitors (ACEI), angiotensin receptor blockers (ARB), diuretics, and statins were continued based on patients' clinical status.

### 2.4. Follow-Up

All patients were evaluated at the outpatient clinic once a week during a three-month follow-up period after RFCA. Clinical symptoms including palpitations, chest tightness, shortness of breath, and fatigue were recorded. All patients' electrocardiogram (ECG) recordings were noted during every visit and when any uncomfortable symptoms occurred. Moreover, 24-hour Holter monitoring was carried out after RFCA and every month. Laboratory parameters including liver function, renal function, and thyroid function were tested monthly. Lung CT was carried out at the end of the follow-up period. All patients were encouraged to undergo an ECG immediately when symptoms such as palpitations occurred.

### 2.5. Outcome Events

The primary outcomes were ER- and AAD-associated adverse effects during the blanking period after RFCA. The terminal points of ER were set to record any atrial arrhythmia, including AF, AFL, and AT, on the ECG when clinical symptoms such as palpitations occurred during the follow-up period or without any discomfort; atrial arrhythmias lasting ≥30s were recorded by an ECG or 24-hour Holter monitoring at the outpatient visit. The main outcome variables of AAD-associated adverse effects were abnormal laboratory indicators and specific clinical symptoms caused by amiodarone or propafenone. The main adverse effects of amiodarone were (I) cardiovascular system—sinus bradycardia, sinus arrest, sinoatrial block, atrioventricular block, prolonged QT interval, and torsade de pointes ventricular tachycardia; (II) thyroid function—hyperthyroidism or hypothyroidism; (III) digestive system—constipation, nausea, vomiting, etc.; and (IV) respiratory system—mainly manifested as pulmonary interstitial and alveolar fiber pneumonia, common in long-term and large-scale applications [11]. The main adverse effects of propafenone were (I) the cardiovascular system—sinus bradycardia and atrioventricular block—and (II) the digestive system—similar to amiodarone [15].

## 3. Statistical Analyses

All statistical analyses were performed utilizing SPSS version 20.0 (SPSS, Chicago, Illinois, USA). The 492 patients who met the inclusion criteria were allocated to the original amiodarone group (*n* = 187) or the original propafenone group (*n* = 305) according to the choice of AADs during the blanking period. Continuous variables are expressed as means ± standard deviations or medians (interquartile range) and were compared using Student's *t*-test or Mann–Whitney *U* test as appropriate. Categorical variables are expressed as percentages and were compared using the chi-squared test. All probability values reported were 2-sided, and a probability value < 0.05 was considered statistically significant.

Outcome events in the amiodarone and propafenone groups were compared using the PS framework. The PS approach aims to create a new dataset in which the probability of receiving amiodarone or propafenone is equal (as in a pure randomized trial) to balance patients' baseline characteristics [16]. As shown in Table 1, before PSM, differences between the original amiodarone group and the original propafenone group in 12 variables, which were age, gender, type of AF, complicated with AT before RFCA, hypertensive heart disease (HHD), coronary artery disease (CAD), dilated cardiomyopathy (DM), peripheral vascular disease (PVD), hyperlipemia (HLP), low-density lipoprotein cholesterol (LDL-C), N terminal pro B type natriuretic peptide (NT-proBNP), and creatinine (CR), were statistically significant (*p* < 0.05). Differences in seven variables, which were complicated with valvular heart disease (VHD) before RFCA, diabetes, stoke/transient ischemic attack (TIA), hyperuricemia (HUA), total cholesterol (TC), and using *β*-blockers or statins, between the two groups were obvious (0.05 < *p* < 0.2). Nineteen of the clinical variables above with a certain difference (*p* < 0.2) were selected to match the two groups. Propensity scores were obtained by utilizing logistic regression. Logit-transformed propensity scores matched to the nearest neighbor in a 1 : 1 fashion with a caliper of 0.02 were used for the matching. After PSM, 135 patients were selected from the original amiodarone group to create the new amiodarone group, and this was repeated with the propafenone group. By comparing the data of the matched amiodarone and propafenone groups by using univariate analysis, no significant difference was found in baseline characteristics. Subsequently, clinical endpoints in the amiodarone and propafenone groups were compared within the matched dataset. Absence of ER in the two groups was calculated by utilizing Kaplan–Meier survival analysis with a log-rank test. Multiple clinical variables were evaluated for their association with ER in the univariate analysis. Variables demonstrating a significant association with ER in the univariate analysis were then analyzed in a Cox proportional hazards regression model.

## 4. Results

### 4.1. Baseline Characteristics

The baseline characteristics of each group are presented in Table 1. Univariate analysis revealed that 15 factors were equal in the two original groups (*p* > 0.05). Differences in 12 factors were statistically significant (*p* < 0.05). After PSM, variables of background characteristics were equal in the matched amiodarone group (*n* = 135) and propafenone group (*n* = 135). In the following sections, all statistical comparisons were based on the matched population.

### 4.2. Comparison of ER

**Figure 2:**
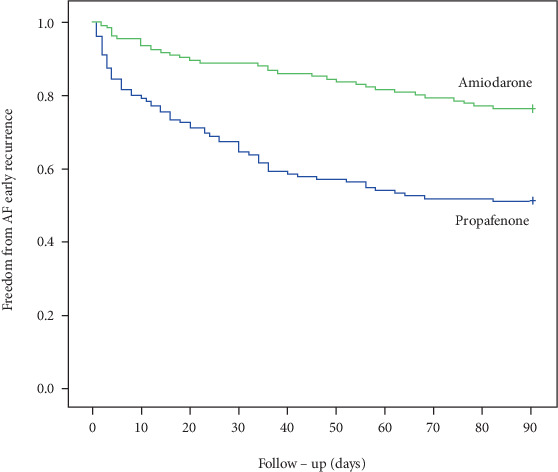
Kaplan–Meier curve analysis of the incidence of ER.

As shown in Table 2, 32 patients (23.7%) in the amiodarone group and 66 patients (48.9%) in the propafenone group experienced atrial tachyarrhythmia during the blanking period after RFCA. The incidence rate of ER was higher in the propafenone group than in the amiodarone group (*p* < 0.001). Kaplan–Meier survival analysis demonstrated that patients in the amiodarone group were more likely to be free from ER than those in the propafenone group (*p* < 0.010, Figure 2).

The matched patients were divided into two groups on the basis of ER: the maintained sinus rhythm group (ER-) and the ER group (ER+). The univariate analysis shown in Table 3 revealed that complicated with VHD (*p* = 0.040), LAD (*p* = 0.019), LVEF (*p* = 0.026), using ACEI/ARB (*p* = 0.024), and treatment with amiodarone after RFCA (*p* < 0.001) were associated with ER in the blanking period.

A multivariable Cox proportional hazards model adjusted complicated with VHD (*p* = 0.040), LAD (*p* = 0.019), LVEF (*p* = 0.026), using ACEI/ARB (*p* = 0.024), and treatment with amiodarone after RFCA demonstrated that treatment with amiodarone in the blanking period was significantly associated with a lower ER incidence rate than propafenone (HR = 0.416, 95% CI 0.272–0.637, *p* < 0.001) (Table 4).

### 4.3. Comparison of AAD-Associated Adverse Effects

As shown in Table 5, during the three-month follow-up period after RFCA, five patients experienced side effects caused by amiodarone or propafenone. Univariate analysis showed that no significant difference was observed between the two groups (*p* = 0.652).

## 5. Discussion

The novel finding of the present study is that in the blanking period, ablated AF patients who took amiodarone had a lower risk of ER than those who took propafenone. Our study's strengths include the full investigation of the population characterized in detail and the propensity matching analyses to control for confounders in the evaluation of patients' outcomes.

As previously reported, ER is common but not represented to the failure of RFCA [7, 17]. It is currently believed that the mechanisms of ER, LR, and long-term recurrence after RFCA are not identical. It has been reported that ablation-related inflammation is the main mechanism of ER. Local inflammation and myocardial tissue edema caused by RFCA may trigger atrial tachyarrhythmia [18]. Badger et al. found that a stable ablation-related scar formed within three months after RFCA, indicating that atrial tachyarrhythmia was more likely to occur in the blanking period [19]. The findings of Lim et al. demonstrated that elevated inflammatory markers, including high-sensitivity CRP and troponin-T, could predict ER within three days after RFCA [20]. Furthermore, catheter ablation leads to autonomic dysregulation, causing increased sympathetic tone and decreased parasympathetic tone, which may lead to tachyarrhythmia [5, 21]. ER after RFCA is currently recognized as an independent predictor of LR. Mugnai et al. found that ER was independently associated with LR (HR = 6.79) [22]. Moreover, Pieragnoli et al. demonstrated that 82.3% of patients with ER after RFCA experienced LR, whereas only 30.2% of patients without ER experienced LR [23]. Yalin et al. found that arrhythmia recurrence during the blanking period was an independent predictor of long-term AF recurrence, although the treatment was not RFCA but second-generation cryoballoon [24]. Therefore, preventing ER could help in reducing LR.

It is currently believed that short-term application of AADs in the blanking period can reduce the risk of ER effectively. A meta-analysis conducted by Xu et al. showed that postprocedural temporary administration of AADs in patients after RFCA reduced ER of AF (OR = 0.47) [8]. The EAST AF trial showed that patients assigned to AAD were associated with a significantly higher event-free rate from recurrent atrial tachyarrhythmias during the blanking period. It also found that there was no significant difference in the one-year event-free rate from recurrent atrial tachyarrhythmias between the groups [25]. However, AAD treatment after ablation can help maintain sinus rhythm and promote benign electrical reconstruction of the atrium. Therefore, the 2016 ESC Guidelines for the management of atrial fibrillation developed in collaboration with EACTS recommended the use of AADs during the blanking period after AF ablation [7]. However, there is no definitive conclusion on the choice of AAD. Propafenone and amiodarone are common and effective in maintaining sinus rhythm [7, 10]. We found that the ER incidence rate in the amiodarone group was lower than that in the propafenone group (23.7% vs 48.9%). Univariate analysis and Cox proportional hazards analysis of ER showed that the amiodarone group had a lower risk of atrial arrhythmia recurrence than the propafenone group in the blanking period. Based on the pharmacological effects of amiodarone, we reasonably speculate that its mechanism of reducing ER is as we have outlined below. First, it has been confirmed that amiodarone can inhibit calcium channel blockers, which was related to its anti-inflammatory effect [13]. Second, amiodarone is an inhibitor of phospholipases A1, A2, and C, which acts in the first step of the production of inflammatory mediators in the arachidonic acid metabolism [26]. Third, it has been reported that amiodarone has noncompetitive antisympathetic effects that relieve the autonomic dysfunction of the heart caused by ablation during the blanking period by inhibiting the sympathetic nerve [27]. Finally, previous studies have shown that amiodarone is a multi-ion channel blocker which can prolong the action potential duration and effective refractory period more effectively than propafenone [12, 28]. In summary, amiodarone plays a unique role in reducing the incidence rate of ER during the blanking period after RFCA.

It is traditionally believed that amiodarone may lead to extracardiac side effects, especially in long-term therapy, which makes it a second-line treatment in patients who are able to take propafenone [7]. In the present study, there was no significant difference in AAD-associated adverse effects between the amiodarone group and the propafenone group in the blanking period (*p* = 0.652). Furthermore, we found that no serious adverse effects such as malignant arrhythmia or pulmonary fibrosis occurred in any group during the follow-up period. These results may indicate that the short-term application of amiodarone is as safe as that of propafenone. However, monitoring adverse reactions requires larger study populations and longer follow-up periods, as it is difficult to detect significant differences in adverse effects between amiodarone and propafenone in the blanking period, particularly for thyroid function and pulmonary fibrosis, whose occurrence is time-dependent [11].

The current consensus is that propafenone is suitable for patients without significant ischemic heart disease, significant left ventricular hypertrophy, and heart failure [15]. Although 14.8% of patients were complicated with CAD in the propafenone group, we had excluded patients with acute or old myocardial infarction, unstable angina, and heart failure. Furthermore, we did not include patients who took other effective AADs, including sotalol, dronedarone, and flecainide, in the blanking period due to the lack of such a population. If such patients had been included, the present study would have been more conclusive.

## 6. Limitations

First, it was a single-center retrospective study which was not randomized and had selection bias. Second, a few patients were asymptomatic when atrial arrhythmias occurred. Third, 202 patients were excluded (29.1%) by the exclusion criteria, which was a high rate of rejection.

## 7. Conclusions

Treatment with amiodarone in the blanking period is more effective in reducing ER than propafenone. In our research, there was no significant difference in safety between amiodarone and propafenone, which emphasized the short-term application of AADs during the blanking period.

## Figures and Tables

**Table 1:** Baseline characteristics of population.

Variable	Original amiodarone group (*n* = 187)	Original propafenone group (*n* = 305)	*p*	Amiodarone group (*n* = 135)	Propafenone group (*n* = 135)	*p*
General information						
Age, years^+^	63 (54-67)	59 (52-65)	0.018	63 (54−67)	62 (56−69)	0.398
Sex, M/F^+^	132/55	169/136	0.001	92/43	84/51	0.307
BMI, kg/m^2^	24.4 (22.5 − 26.8)	24.3 (22.0 − 26.4)	0.258	24.4 (22.5 − 26.1)	24.3 (22.4 − 26.4)	0.943
AF type						
PAF/PeAF^+^	138/49	290/15	<0.001	118/17	120/15	0.706
Medical history						
FL	21 (11.2)	37 (12.1)	0.763	17 (12.6)	11 (8.1)	0.231
AT^+^	19 (10.2)	58 (19.0)	0.009	15 (11.1)	16 (11.9)	0.849
HT	92 (49.2)	133 (43.6)	0.227	67 (49.6)	74 (54.8)	0.394
HHD^+^	53 (28.3)	55 (18.1)	0.003	36 (26.7)	40 (29.6)	0.588
CAD^+^	29 (15.5)	23 (7.5)	0.005	18 (13.3)	20 (14.8)	0.726
DM^+^	8 (4.3)	4 (1.5)	0.038	3 (2.2)	4 (2.9)	0.702
VHD^+^	27 (14.4)	30 (9.9)	0.122	19 (14.1)	18 (13.1)	0.860
GUCH	9 (4.8)	11 (3.7)	0.511	5 (3.7)	4 (3.0)	0.735
Diabetes^+^	37 (19.8)	41 (13.4)	0.061	19 (14.1)	25 (18.5)	0.323
Stoke/TIA^+^	35 (18.7)	38 (12.5)	0.058	22 (16.3)	27 (20.0)	0.430
PVD^+^	8 (4.3)	4 (1.3)	0.038	3 (2.2)	3 (2.2)	1.000
HLP^+^	57 (30.5)	122 (40.0)	0.033	39 (28.9)	45 (33.3)	0.430
HUA^+^	48 (25.7)	59 (19.4)	0.099	30 (22.2)	29 (21.5)	0.863
Laboratory test results						
TC^+^, mmol/L	4.37 ± 1.05	4.51 ± 1.04	0.135	4.33 ± 1.01	4.33 ± 1.04	0.969
LDL-C^+^, mmol/L	2.76 ± 0.92	2.95 ± 0.95	0.027	2.77 ± 0.93	2.72 ± 0.95	0.695
NT-proBNP^+^, ng/L	187.0 (61.0−567.0)	92.0 (46.0−212.0)	<0.001	123.0 (53.0−440.0)	132.0 (60.0−409.0)	0.767
CR^+^, umol/L	78.4 ± 17.0	74.8 ± 16.1	0.011	78.1 ± 16.6	76.6 ± 14.5	0.434
Echocardiogram parameters						
LAD, mm	36.77 ± 5.91	36.78 ± 5.95	0.990	36.92 ± 6.00	35.76 ± 6.60	0.136
LVEF, %	66.1 ± 7.4	66.3 ± 7.3	0.740	65.8 ± 7.3	66.8 ± 6.9	0.248
CHA_2_DS_2_VASc	1.88 ± 1.62	1.62 ± 1.40	0.069	1.81 ± 1.50	2.01 ± 1.49	0.239
Medication use after RFCA						
*β*-Blockers^+^	32 (17.0)	39 (12.9)	0.185	21 (15.6)	22 (16.3)	0.868
ACEI/ARB	56 (29.9)	91 (29.9)	1.000	40 (29.6)	55 (40.7)	0.056
Statins^+^	55 (29.4)	110 (36.1)	0.129	40 (29.6)	46 (34.1)	0.433

+PSM variables. Values are mean ± SD, medians (interquartile range), *n*_1_/*n*_2_ or *n* (%). BMI = body mass index; PAF = paroxysmal atrial fibrillation; PeAF = persistent atrial fibrillation; HT = hypertension; GUCH = grown up with congenital heart disease; LAD = left atrial diameter; LVEF = left ventricular ejection fraction.

**Table 2:** Comparison of ER.

	Amiodarone group *n* = 135	Propafenone group *n* = 135	*p*
ER	32 (23.7)	66 (48.9)	<0.001

Values presented as *n* (%).

**Table 3:** Univariate analysis of ER.

Variable	ER+	ER-	*p*
General information			
Age, years	62.30 ± 9.08	60.46 ± 9.50	0.122
Sex, M/F	59/39	117/55	0.195
BMI, kg/m^2^	24.32 ± 3.33	24.66 ± 3.32	0.424
AF type			
PAF/PeAF	87/11	151/21	0.810
Medical history			
AFL	10 (10.2)	18 (10.5)	0.946
AT	12 (12.2)	19 (11.0)	0.766
HT	54 (55.1)	87 (50.6)	0.475
HHD	26 (26.5)	50 (29.1)	0.656
CAD	17 (17.3)	21 (12.2)	0.243
DM	1 (1.0)	6 (3.5)	0.220
VHD^∗^	19 (19.4)	18 (10.5)	0.040
GUCH	3 (3.1)	6 (3.5)	0.851
Diabetes	16 (16.3)	28 (16.2)	0.992
Stoke/TIA	2 (2.1)	7 (4.1)	0.220
PVD	2 (2.1)	4 (2.3)	0.879
HLP	32 (32.7)	52 (30.3)	0.684
HUA	17 (17.3)	42 (24.4)	0.176
Laboratory test results			
TC, mmol/L	4.27 ± 1.01	4.37 ± 1.03	0.422
LDL-C, mmol/L	2.69 ± 0.97	2.78 ± 0.92	0.425
NT-proBNP, ng/L	370.07 ± 20.12	334.57 ± 21.32	0.390
CR, umol/L	77.07 ± 15.53	77.46 ± 15.62	0.845
Echocardiogram parameters			
LAD^∗^, mm	37.02 ± 6.41	35.15 ± 6.03	0.019
LVEF^∗^, %	67.72 ± 7.49	65.50 ± 6.85	0.026
CHA_2_DS_2_VASc	2.01 ± 1.39	1.86 ± 1.55	0.430
Medication use after RFCA			
*β*-Blockers	13 (13.3)	30 (17.4)	0.367
ACEI/ARB^∗^	43 (43.8)	52 (30.2)	0.024
Statins	32 (32.7)	54 (31.4)	0.831
Amiodarone^∗^	32 (32.7)	103 (59.8)	<0.001

^∗^Variables associated with ER statistically (*p* < 0.05). Values are mean ± SD, *n*_1_/*n*_2_ or *n* (%).

**Table 4:** Multivariable Cox proportional hazards analysis of ER.

Variable	Multivariable Cox proportional hazards analysis		
	HR	95% CI	*p*
VHD	1.641	0.980−2.750	0.060
LAD	0.972	0.942−1.003	0.079
LVEF	1.028	0.998−1.059	0.067
ACEI/ARB	1.400	0.931−2.106	0.106
Amiodarone^∗^	0.416	0.272−0.637	<0.001

∗p < 0.05.

**Table 5:** Comparison of AAD-associated adverse effects.

	Amiodarone group *n* = 135	Propafenone group *n* = 135	*p*
AAD-related adverse effects	3 (2.2)	2 (1.5)	0.652
Bradycardia	1 (0.7)	2 (1.5)	0.562
Prolonged QT interval	1 (0.7)	None	0.316
Thyroid dysfunction	1 (0.7)	None	0.316

Values presented as *n* (%).

## Data Availability

The datasets used or analyzed during the present study are available from the corresponding author on reasonable request.

## References

[1] Nielsen J. C., Johannessen A., Raatikainen P. (2017). Long-term efficacy of catheter ablation as first-line therapy for paroxysmal atrial fibrillation: 5-year outcome in a randomised clinical trial. *Heart (British Cardiac Society)*.

[2] Mont L., Bisbal F., Hernández-Madrid A. (2014). Catheter ablation vs. antiarrhythmic drug treatment of persistent atrial fibrillation: a multicentre, randomized, controlled trial (SARA study). *European heart journal*.

[3] Olshausen G., Uijl A., Jensen‐Urstad M. (2020). Early recurrences of atrial tachyarrhythmias post pulmonary vein isolation. *Journal of cardiovascular electrophysiology*.

[4] Yanagisawa S., Inden Y., Kato H. (2016). Effect and significance of early reablation for the treatment of early recurrence of atrial fibrillation after catheter ablation. *The American journal of cardiology*.

[5] Liang J. J., Dixit S., Santangeli P. (2016). Mechanisms and clinical significance of early recurrences of atrial arrhythmias after catheter ablation for atrial fibrillation. *World Journal of Cardiology*.

[6] Mujović N., Marinković M., Marković N. (2018). The relationship of early recurrence of atrial fibrillation and the 3-month integrity of the ablation lesion set. *Scientific Reports*.

[7] Kirchhof P., Benussi S., Kotecha D. (2016). 2016 ESC guidelines for the management of atrial fibrillation developed in collaboration with EACTS. *European heart journal*.

[8] Xu X., Alida C. T., Yu B. (2015). Administration of antiarrhythmic drugs to maintain sinus rhythm after catheter ablation for atrial fibrillation: a meta-analysis. *Cardiovascular therapeutics*.

[9] Calkins H., Hindricks G., Cappato R. (2018). 2017 HRS/EHRA/ECAS/APHRS/SOLAECE expert consensus statement on catheter and surgical ablation of atrial fibrillation: executive summary. *EP Europace*.

[10] January C. T., Wann L. S., Calkins H. (2019). 2019 AHA/ACC/HRS focused update of the 2014 AHA/ACC/HRS guideline for the management of patients with atrial fibrillation: a report of the American College of Cardiology/American Heart Association Task Force on Clinical Practice Guidelines and the Heart Rhythm Society in collaboration with the Society of Thoracic Surgeons. *Circulation*.

[11] Vassallo P., Trohman R. G. (2007). Prescribing amiodarone: an evidence-based review of clinical indications. *Journal of the American Medical Association*.

[12] Freemantle N., Lafuente-Lafuente C., Mitchell S., Eckert L., Reynolds M. (2011). Mixed treatment comparison of dronedarone, amiodarone, sotalol, flecainide, and propafenone, for the management of atrial fibrillation. *EP Europace*.

[13] Halici Z., Dengiz G. O., Odabasoglu F., Suleyman H., Cadirci E., Halici M. (2007). Amiodarone has anti-inflammatory and anti-oxidative properties: an experimental study in rats with carrageenan-induced paw edema. *European journal of pharmacology.*.

[14] January C. T., Wann L. S., Alpert J. S. (2014). 2014 AHA/ACC/HRS guideline for the management of patients with atrial fibrillation: a report of the American College of Cardiology/American Heart Association Task Force on Practice Guidelines and the Heart Rhythm Society. *Journal of the American College of Cardiology*.

[15] Stoschitzky K., Stoschitzky G., Lercher P., Brussee H., Lamprecht G., Lindner W. (2016). Propafenone shows class Ic and class II antiarrhythmic effects. *EP Europace*.

[16] Austin P. C. (2011). An introduction to propensity score methods for reducing the effects of confounding in observational studies. *Multivariate Behavioral Research*.

[17] Lellouche N., Jaïs P., Nault I. (2008). Early recurrences after atrial fibrillation ablation: prognostic value and effect of early reablation. *Journal of cardiovascular electrophysiology*.

[18] Miyazaki S., Taniguchi H., Nakamura H. (2015). Clinical significance of early recurrence after pulmonary vein antrum isolation in paroxysmal atrial fibrillation - insight into the mechanism. *Circulation Journal*.

[19] Badger T. J., Oakes R. S., Daccarett M. (2009). Temporal left atrial lesion formation after ablation of atrial fibrillation. *Heart Rhythm*.

[20] Lim H. S., Schultz C., Dang J. (2014). Time course of inflammation, myocardial injury, and prothrombotic response after radiofrequency catheter ablation for atrial fibrillation. *Circulation. Arrhythmia and Electrophysiology*.

[21] Nalliah C. J., Lim T. W., Kizana E. (2015). Clinical significance of early atrial arrhythmia type and timing after single ring isolation of the pulmonary veins. *EP Europace*.

[22] Mugnai G., de Asmundis C., Hünük B. (2016). Second-generation cryoballoon ablation for paroxysmal atrial fibrillation: predictive role of atrial arrhythmias occurring in the blanking period on the incidence of late recurrences. *Heart Rhythm*.

[23] Pieragnoli P., Perini A. P., Ricciardi G. (2017). Recurrences in the blanking period and 12-month success rate by continuous cardiac monitoring after cryoablation of paroxysmal and non-paroxysmal atrial fibrillation. *Journal of cardiovascular electrophysiology*.

[24] Yalin K., Abdin A., Lyan E. (2018). Safety and efficacy of persistent atrial fibrillation ablation using the second-generation cryoballoon. *Clinical Research in Cardiology*.

[25] Kaitani K., Inoue K., Kobori A. (2016). Efficacy of antiarrhythmic drugs short-term use after catheter ablation for atrial fibrillation (EAST-AF) trial. *European heart journal*.

[26] Ozbakis-Dengiz G., Halici Z., Akpinar E., Cadirci E., Bilici D., Gursan N. (2007). Role of polymorphonuclear leukocyte infiltration in the mechanism of anti-inflammatory effect of amiodarone. *Pharmacological reports: PR*.

[27] Kodama I., Kamiya K., Toyama J. (1999). Amiodarone: ionic and cellular mechanisms of action of the most promising class III agent. *The American journal of cardiology*.

[28] Kochiadakis G. E., Igoumenidis N. E., Hamilos M. E., Marketou M. E., Chlouverakis G. I., Vardas P. E. (2007). A comparative study of the efficacy and safety of *procainamide* versus *propafenone* versus *amiodarone* for the conversion of recent-onset atrial fibrillation. *The American journal of cardiology*.

